# Do childhood depressive symptoms interfere with intelligence in adulthood?

**DOI:** 10.11606/s1518-8787.2023057004918

**Published:** 2023-09-14

**Authors:** Janielle Ferreira de Brito Lima, Raina Jansen Cutrim Propp Lima, Mônica Araújo Batalha, Antônio Augusto Moura da Silva, Marizélia Rodrigues Costa Ribeiro, Rosângela Fernandes Lucena Batista

**Affiliations:** I Universidade Federal do Maranhão Departamento de Saúde Pública São Luís MA Brazil Universidade Federal do Maranhão. Departamento de Saúde Pública. São Luís, MA, Brasil. São Luís, MA, Brazil.

**Keywords:** Child, Adult, Depression, Intelligence

## Abstract

**OBJECTIVE:**

To investigate the effects of depressive symptoms in childhood on the intellectual development of young adults.

**METHODS:**

Study conducted with a birth cohort of São Luís, Maranhão, Brazil, composed of 339 participants evaluated between 7 and 9 years and between 18 and 19 years. Structural equation modeling (young adult education, sex, race/color) and childhood variables (nutritional status, depressive symptoms, cognitive function, head of household’s and mother’s education, family income) were used. In addition, head of household’s occupation, mother’s age, and presence of partner were tested as determinants of adults’ intelligence quotient (IQ).

**RESULTS:**

Presence of depressive symptoms in childhood triggered a reduction of 0.342 in standard deviation (SD) and -3.83 points in the average IQ of adults (p-value < 0.001). Cognitive function in childhood had a total and direct positive effect (standardized coefficient [SC] = 0.701; p-value < 0.001) on IQ, increasing 7.84 points with each increase in level. A positive indirect effect of child nutritional status (SC = 0.194; p-value = 0.045), head of household’s (SC = 0.162; p-value = 0.036), and mother’s education was identified, the latter mediated by cognitive function in childhood (SC = 0.215; p-value = 0.012) on the IQ of young people.

**CONCLUSION:**

Presence of depressive symptoms in childhood triggered a long-term negative effect on intelligence, reducing the IQ score in adulthood.

## INTRODUCTION

The development of cognitive skills, mainly represented by intelligence and academic performance, has been widely studied due to its impact on socially relevant outcomes such as leadership ability, success at work, and social life^1,2^.

Although intelligence is strongly influenced by heredity, parental stimulation, education, and nutritional and psychological status are known to also affect its development^3^. Among these variables, the mental health of children and adolescents has been the subject of many studies^4^ due to the high worldwide prevalence of mental disorders in this age group in recent years, especially depression, which was identified in 6.2% of individuals aged 5 to 17 in 38 countries^4^. In Brazil, depressive disorders have been the most prevalent among the mental problems that affect children and adolescents^5,8^.

There is evidence that symptoms of internalization and cognitive ability are interrelated variables in child development until adolescence^6^. However, a study conducted with Canadian children did not find significant evidence that the presence of internalization symptoms interfered with academic performance in adolescence, which is directly related to intellectual development. The only significant effect was a positive association of internalization with academic performance from 4 to 11 years of age^9^.

Changes caused by depressive symptoms usually cause significant damage to a child’s life, affecting their behavior at home, at school and with friends. The drop in school performance is one of the first indicators of depression in children, in addition to the development of dysphoria, isolation, and sadness^10^.

It remains unclear how depressive symptoms can interfere with intellectual development until adulthood, with the pathways of association between these variables, and how one variable influences another. It is also necessary to understand if the difficulties of one cause difficulties in another or if they relate only because they share causes.

Thus, the objective of this study is to answer the following questions: does the presence of depressive symptoms in childhood have a direct effect on the adult’s intelligence? Do parents’ socioeconomic variables during childhood have a greater effect on the adult’s intelligence than the presence of depressive symptoms in childhood? Is the association between childhood depressive symptoms and adult intelligence mediated by childhood cognition?

## METHODS

### Study Design

This is a cohort study conducted with individuals born in the city of São Luís, Maranhão, Brazil, involving two different periods: childhood and adulthood. This cohort is part of the research *Determinantes ao longo do ciclo vital da obesidade, precursores de doenças crônicas, capital humano e saúde mental* (Determinants throughout the life cycle of obesity, precursors of chronic diseases, human capital and mental health), developed by the Universidade Federal do Maranhão (UFMA), Faculdade de Medicina de Ribeirão Preto da Universidade de São Paulo (FMRP/USP) and the Universidade Federal de Pelotas (UFPel)..

### Study Population and Sample

The first phase of the cohort was initiated at birth in 10 public and private hospitals in the city, from March 1997 to February 1998, including 96.3% of births of the period through systematic sampling with proportional stratification according to the number of births in each maternity in one out of seven deliveries. Multiple births, stillbirths, and twin births were excluded. The final sample totaled 2,443 births^11^.

The second phase occurred when the children were 7 to 9 years old, in 2005 and 2006, through a complex sampling design, using the variable birth weight to define the sample necessary for evaluation at school age. The final sample totaled 805 children in this phase, 673 being followed since birth and 132 children born between 1997 and 1998 included in the retrospective cohort^8^.

The third phase occurred from January to November 2016, when the subjects were 18–19 years of age. Of the 805 participants from the previous stage, 339 participated in this follow-up. At this stage, we investigated socioeconomic and demographic status, income, lifestyle habits, cognitive skills, mental illness, among others^12^. The study included subjects who, at the time of evaluation, underwent tests of mental health, cognitive function in childhood and intellectual development in adulthood.

### Data Collection

In the school phase, a standardized questionnaire was applied to parents or guardians of children containing demographic questions. Weight and height were measured, with the children barefoot and in light clothing, using a periodically calibrated precision scale and an anthropometer^8^. To assess cognitive function in childhood, the Raven’s Colored Progressive Matrices (RCPM) was applied, which assesses general aspects of intelligence^13^; and the Draw-a-Person test (DAP test), which assesses emotional maturity and psychomotor development^14^. To assess the presence of depressive symptoms in childhood, the Children’s Depression Inventory was used^15^.

Adult intelligence was assessed by the Wechsler Adult Intelligence Scale (WAIS III), which depicts a punctual measure of intelligence level. To verify the results, the raw data of the verbal and execution scales were initially analyzed. From the sum of scores of all subtests, the crude results were converted into weighted results and analyzed by the table corresponding to the WAIS III manual. The total intelligence quotient (IQ) was obtained by summing the raw values of the verbal and execution scales, and analyzing the values in the table by age, reflecting the participants’ intelligence levels^16^. In cases where the participant had any condition that made it impossible to perform the test, it was not applied.

### Variables

The dependent variable was the IQ of the young adult, obtained through the application of WAIS-III. For the theoretical model tested, the variable was treated as continuous numerical. For classification purposes, it can be categorized as: lower (≤ 89 points); medium (90 to 109 points); higher (≥ 110 points)^16^.

The explanatory variables observed in childhood were: sex (female; male), race/color (black; mixed/yellow/oriental; white); nutritional status: low weight (< 17 kg/m^2^) or adequate (> 17 kg/m^2^)^8^; depressive symptoms: absent (≥ 17 points) or present (< 17 points)^15^; education of the head of household and of the mother in full years (0–4 years; 5–8; 9 or more); family income in minimum wages (continuous numerical); occupation of the head of household (unskilled and unemployed manual; qualified and semi-skilled manual; non-manual); age of the mother (continuous numerical); presence of a partner (no; yes).

The variable cognitive function in childhood was constructed from the variables cognitive function measured by the RCPM test, treated as categorical variable: below average (0–25); average (26–74); above average (75–100)^13^ and cognitive function measured by the DAP test, treated as categorical variable: below average (< 25^th^ percentile); average (25^th^ percentile); above average (> 25^th^ percentile)^14^. The education variable of the young adult was treated as categorical (0–4 years; 5–8; 9 or more).

### Statistical Analysis

The descriptive analysis of the data was performed using version 14 of the Stata program (StataCorp., CollegeStation, United States of America). Categorical variables were presented through absolute and relative frequencies, numerical variables by mean and standard deviation.

Structural equation modeling was used^17^ to investigate the association of depressive symptoms in childhood with covariates and their effects on intelligence in adulthood. A hybrid model was built, composed of confirmatory factor analysis used to construct the latent variable cognitive function in childhood and pathway analysis used to analyze the effects of depressive symptoms in childhood on the IQ in adulthood and estimate the linear relationships between the variables. For this, version 7 of the Mplus software was used.

According to the proposed theoretical model, the family socioeconomic variables observed during the participant’s childhood, occupied the most distal position and indicated the presence of depressive symptoms, nutritional status and cognitive function of the child, which determined the education of the young adult and their IQ (Figure).

**Figure:**
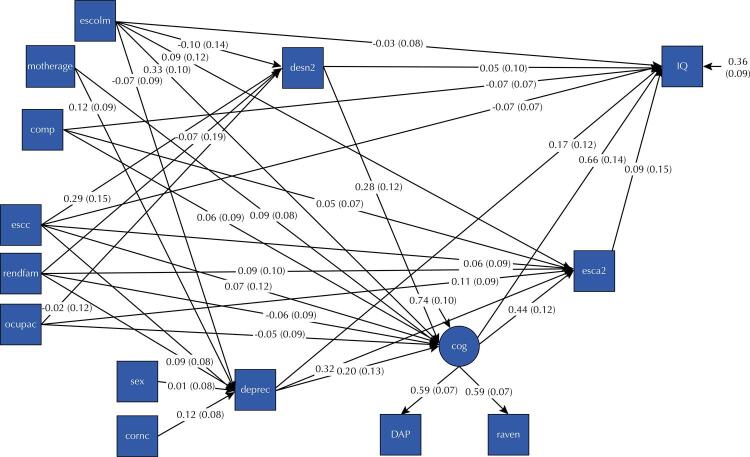
Path diagram, with standardized coefficients, of the association of depressive symptoms in childhood with the intelligence quotient of young adults in the São Luís RPS Consortium Cohort. São Luís (Maranhão), Brazil, 2004–2016.

cog: childhood cognition; comp: presence of a partner; cornc: race/color of the child; deprec: depressive symptoms in childhood; desn2: malnutrition; DAP: cognitive development in childhood measured by the DAP test; esca2: level of education of the young adult; escc: education of the head of household during childhood; escolm: maternal education during childhood; motherage: age of the child’s mother; ocupac: occupation of the head of household during childhood; IQ: intelligence quotient of the young adult; raven: cognitive development in childhood measured by the Raven’s colored progressive matrices test; rendfam: family income during childhood; sex: sex.

The estimator used was the robust weighted least squares mean and variance adjusted (WLSMV), used for analysis of the observed categorical variables. Theta parameterization (θ) was used to control differences in residual variances.

The missing data were imputed to the variables by Mplus software, based on the variables prior to the parameterization in the theoretical model, using frequency and Bayesian analyses^18^. With this imputation, the missing data did not hinder the result.

To evaluate the model fit, the following was considered as good: a) p-value > 0.05 for the Chi-square test (χ^2^); b) root mean square error of approximation (RMSEA) value < 0.05 and upper limit of the 90% confidence interval < 0.08; c) values greater than 0.95 for the comparative fit index (CFI) and the Tucker-Lewis index (TLI); and d) weighted root mean square residual (WRMR) values < 1^17^.

In the analysis of the standardized estimates for the construction of the latent variable cognition in childhood, factor load > 0.5 was considered with a p-value < 0.05 as indicative that the correlation between the observed variable and the construct is moderately high in magnitude^17^.

The *modindices* command was used to obtain suggestions for changes in the initial hypotheses^18^, however, there were no plausible proposals from a theoretical point of view. In the final model, the total, direct, and indirect effects of the latent and observed variables were evaluated. An effect was considered when p-value was < 0.05.

To interpret the effects of the variables on the IQ of young adults, the value of the standardized coefficient of the total effect obtained in the structural model was multiplied by the standard deviation of the IQ.

### Ethical Aspects

The study met the criteria of Resolution^19^ 466/2012of the National Health Council of Brazil. The subjects’ guardians signed an informed consent form (ICF). Withdrawal was allowed without any prejudice to the interviewee at any stage of the research. The projects and the ICFs of both phases of the research were approved by the Research Ethics Committee of the Hospital Universitário da Universidade Federal do Maranhão: Consolidated Opinion No. 60, of April 18, 2005, and Consolidated Opinion No. 1,302,489, of October 29, 2015.

## RESULTS

In the sample of 339 young adults, 55.16% were female, 67.85% were mixed/yellow/oriental and 83.78% had 5 to 8 years of study (Table 1). The mean IQ of the participants was 99.45 (11.19) points. The majority (60.47%) achieved results classified as average, with the lowest performance being 70 and the highest 137 points.

**Table 1:** Socioeconomic, demographic, and family characteristics, presence of depressive symptoms and cognitive development and intelligence quotient of young adults in the São Luís RPS Consortium Cohort. São Luís (Maranhão), Brazil, 2004–2016.

Characteristic	n (%)
Of the child	
Sex	
Female	187 (55.16)
Male	152 (44.84)
Race/color	
Black	30 (8.85)
Mixed/yellow/oriental	230 (67.85)
White	79 (23.30)
Malnutrition^a^	
Yes	25 (7.40)
No	312 (92.31)
Cognitive development (RCPM)^a^	
Below average	88 (25.96)
Average	150 (44.25)
Above average	100 (29.50)
Cognitive development (DAP)^a^	
Below average	102 (30.09)
Average	192 (56.64)
Above average	42 (12.68)
Depressive symptoms^a^	
Absent	277 (81.71)
Present	62 (18.29)
Of the family during childhood	
Mother’s education (years)^a^	
0–4	45 (13.27)
5–8	89 (26.25)
≥ 9	198 (58.41)
Presence of partner	
No	70 (20.71)
Yes	269 (79.29)
Education of the head of household (years)^a^	
0–4	106 (31.27)
5–8	71 (20.94)
≥ 9	143 (42.18)
Family income (in minimum wages)^a^	
< 1	66 (19.47)
1 < 4	230 (67.85)
≥ 4	27 (7.96)
Occupation of the head of household^a^	
Unskilled manual or unemployed	196 (56.64)
Qualified or semi-qualified manual	100 (29.50)
Non-manual	45 (13.27)
Of the young adult	
Education (years)	
0–4	18 (5.31)
5–8	284 (83.78)
≥ 9	37 (10.91)
Intelligence quotient (IQ)	
Below average	63 (18.58)
Average	205 (60.47)
Above average	71 (20.94)
Total	339 (100)

RCPM: Raven’s colored progressive matrices test; DAP: Draw-a-Person test.

^a^ Excluded the ignored ones.

During childhood, 92.31% of the study participants had adequate nutritional status and 18.29% had depressive symptoms. According to the RCPM test, 44.25% presented average cognitive function in this phase and, according to the DAP, 56.64%. The mean age of the participants’ mothers in this phase was 23.15 (5,22) years. Most of them lived with a partner (79.29%) and had 9 or more years of study (58.41%). Regarding the heads of households, 56.64% were unemployed or engaged in unskilled manual occupation and 42.18% had 9 or more years of education. The income of 67.85% of families was between one and less than four minimum wages (Table 1).

The model proposed to investigate the pathways of the association between the presence of depressive symptoms in childhood and intelligence in adulthood presented a good fit for the indicators RMSEA, CFI, TLI, and WRMR (Table 2) and there were no plausible suggestions for modification.

**Table 2:** Model fit indices for intellectual development of young adults of the São Luís RPS Consortium Cohort. São Luís (Maranhão), Brazil, 2004–2016.

Fit indices	Model^a^
χ^2b^	265,810
Degrees of freedom	63
P-value	< 0.001
RMSEA	0.018
90%CI	0.000–0.049
P-value	0.957
CFI	0.985
TLI	0.968
WRMR	0.608

RMSEA: *root mean square error of approximation*; CFI: *comparative fit index*; TLI: *Tucker-Lewis index*; WRMR: *weighted root mean square residual*; 90% confidence interval.

^a^ Best fit final model.

^b^ Chi-square test.

The factor analysis for the construction of cognitive function in childhood showed that the indicator variables correlated with the construct, presenting a factor load > 0.5 and p values < 0.001 (Table 3).

**Table 3:** Standardized coefficient, standard error, and p-values of the cognition construct in childhood and the direct effects of the indicator variables on the intelligence quotient of young adults in the São Luís RPS Consortium Cohort. São Luís (Maranhão), Brazil, 2004–2016.

Variables	Standardized coefficient	Standard error	p-value
Latent variable			
Cognition in childhood			
Cognitive development (DAP)	0.588	0.073	< 0.001
Cognitive development (RCPM)	0.542	0.065	< 0.001
Direct effects			
Intelligence quotient			
Depressive symptoms in childhood	-0.171	0.122	0.16
Cognition in childhood	0.662	0.141	< 0.001
Malnutrition in childhood	0.046	0.097	0.633
Partner presence	-0.072	0.067	0.28
Maternal education	-0.025	0.079	0.75
Head of household’s education	0.065	0.065	0.319
Young adult’s education	0.088	0.152	0.562
Cognition in childhood			
Depressive symptoms in childhood	0.205	0.128	0.11
Malnutrition in childhood	0.276	0.125	0.027
Mother’s age	0.088	0.079	0.263
Mother’s education	0.325	0.095	0.001
Partner presence	0.059	0.092	0.523
Family income in childhood	-0.06	0.093	0.524
Head of household’s education	0.067	0.122	0.58
Occupation of the head of household	-0.048	0.089	0.592
Depressive symptoms in childhood			
Sex	0.014	0.083	0.861
Race/color	0.12	0.081	0.141
Family income in childhood	0.092	0.081	0.256
Mother’s age	0.123	0.091	0.202
Maternal education	-0.065	0.093	0.484
Head of household’s education	0.116	0.091	0.202
Malnutrition in childhood			
Family income in childhood	-0.066	0.187	0.726
Maternal education	-0.103	0.143	0.475
Head of household’s education	0.291	0.152	0.056
Occupation of the head of household	-0.021	0.118	0.861
Young adult’s education			
Depressive symptoms in childhood	-0.315	0.133	0.018
Cognition in childhood	0.437	0.121	< 0.001
Family income in childhood	0.088	0.104	0.398
Occupation of the head of household	0.109	0.093	0.243
Head of household’s education	0.056	0.093	0.55
Maternal education	0.087	0.124	0.48
Partner presence	0.054	0.073	0.459

RCPM: Raven’s colored progressive matrices test; DAP: Draw-a-Person test.

The presence of depressive symptoms in childhood had a total negative effect of -0.342 SD on the average IQ in adulthood (Table 4), which corresponds to -3.83 points (p-value < 0.001). Cognitive development in childhood had a total and direct positive effect on IQ (Table 4), revealing an increase of 0.701 SD in its average (p-value < 0.001), corresponding to an increase of 7.84 points at each elevation in the level of child cognitive development.

**Table 4:** Standardized coefficient, standard error, and p-values of the total, direct and indirect effects of depressive symptoms, cognition and malnutrition in childhood and levels of education of the mother and of head of household on the intelligence quotient of young adults in the São Luís RPS Consortium Cohort. São Luís (Maranhão), Brazil, 2004–2016.

Pathways	Standardized coefficient	Standard error	p-value
Total, direct, and indirect effects			
Depressive symptoms in childhood			
Total effect	-0.342	0.08	< 0.001
Direct effect	-0.171	0.122	0.16
Indirect effect	-0.171	0.103	0.096
Cognition in childhood			
Total effect	0.701	0.102	< 0.001
Direct effect	0.662	0.141	< 0.001
Indirect effect	0.039	0.062	0.531
Malnutrition in childhood			
Total effect	0.24	0.078	0.002
Direct effect	0.046	0.097	0.633
Indirect effect	0.194	0.097	0.045
Head of household’s education			
Total effect	0.227	0.057	< 0.001
Total effect	0.065	0.065	0.319
Direct effect	0.162	0.077	0.036
Maternal education			
Total effect	0.163	0.056	0.004
Direct effect	-0.025	0.079	0.75
Indirect effect	0.188	0.084	0.026
Via cognition in childhood	0.215	0.086	0.012

The nutritional status of the participant in childhood (SC = 0.240; p-value = 0.002) and the levels of education of the head of household (SC = 0.227; p-value < 0.001) and of the mother of the participant in this phase (SC = 0.163; p-value = 0.004) showed a total positive effect, predominantly indirect, on IQ in adulthood (Table 4). The positive indirect effect of maternal education on IQ was mediated by childhood cognition (SC = 0.215; p-value = 0.012) (Table 4).

## DISCUSSION

In this study, the presence of depressive symptoms in children had negative long-term results in the development of intelligence, reducing the IQ score in adulthood. This effect was not mediated by cognitive function in childhood, whose impact on IQ was direct. Positive indirect effects of the participant’s nutritional status in childhood, levels of education of the mother and of the head of household on the adult IQ score were observed. Additionally, we observed that cognition in childhood seems to mediate the positive effect of maternal education on the IQ score in adulthood.

It is possible that the reduction in IQ observed in young adults who had depressive symptoms in childhood is explained by the impaired ability to think, concentrate, or make decisions that accompany these symptoms in children. In this age group, the drop in school performance is one of the first signs of depression and can be a manifestation of attention deficit^10^. In this study, depressive symptoms in childhood had a direct negative effect on the education of young adults. Another possible explanation is that depressive symptoms detected in childhood can persist and be intensified into adulthood, thus generating lifelong impacts^20^. Previous studies have shown that changes in mental health in childhood and adolescence can compromise academic performance and the level of education achieved, and the latter, in turn, can impact the job opportunities and careers of these individuals^21,22^.

The direct effect of childhood cognition on adult intelligence observed in this study is in line with previous studies, which reveal high cognitive stability throughout life^23,24^. Yu et al.^23^ observed, using a latent construct of intelligence, that there was considerable stability in intelligence over the four periods investigated: childhood, preschool, school, and adolescence. In addition, the longitudinal progression of intelligence from childhood to adolescence was completely mediated by the intelligence of the previous period. Additionally, data from 588 individuals in the Lothian Birth Cohort 1936 revealed that cognitive ability at age 11 was a predictor of cortical thickness approximately 60 years later and was responsible for more than two-thirds of the cross-sectional association between intelligence and cortical thickness in old age^24^.

Several studies have already shown the importance of the family’s socioeconomic situation in development and cognition in childhood and throughout life^25,26^. In this context, education has been used as one of the main indicators of socioeconomic status^25^. A study conducted with data from seven countries (n = 15.297), including Brazil, revealed that the education of parents could be more important than family wealth in shaping the intelligence of their children (4–22 years)^26^.

The relationship between parenting and child intelligence can be explained by both genetic and environmental factors^25,27^. Parents with a higher level of education seem to invest more money and time in their children, resulting in better health and education conditions^28^, as well as offering an intellectually more stimulating environment, which could result in superior performance on some tests^29^. Evidence from low- and middle-income countries indicates that maternal level of education would be an important protective factor for the child well-being and development^30^, with an effect apparently superior to that of paternal level of education^31^. Mothers with higher levels of education seem to provide their children with greater learning opportunities, higher quality interaction and less experience of parental stress^32^. In addition to these facts, maternal stimulation is among the main mechanisms that explain the relationship between maternal education and child development^31,33^.

In agreement with our findings, recent studies show that child development can also be affected by the child’s nutritional conditions^34,35^. Proper nutrition, in a critical period like childhood, is critical for lifelong brain functioning^35^. In addition, children with adequate nutritional status are able to interact better with their caregivers and the environment, fundamental factors for their full development^36^.

A limitation of this study was the loss of follow-up of the subjects, especially during the third phase, due to the difficulty in locating young adults, despite all the search strategies used. With a larger sample, it would be possible to detect other effects of important determinants.

A strong point is the type of study. Cohort studies have advantages in relation to reverse causality and the possibility of monitoring the same population. Another point is the statistical method used: structural equation modeling. This modeling has the advantage of simultaneously dealing with several dependency relationships and can represent latent variables in these relationships, in addition to modeling the measurement error in the estimation process^17^.

This study demonstrated that IQ in adulthood is directly influenced by levels of cognitive development in childhood. During childhood, favorable socioeconomic status can positively influence IQ in adulthood, but the presence of depressive symptoms can negatively influence IQ in adulthood.
