# Supplementary material for: Do childhood depressive symptoms interfere with intelligence in adulthood?
Source: Rev Saude Publica. 2023 Sep 14;57:64. doi: 10.11606/s1518-8787.2023057004918 (PMC10519686; doi:10.11606/s1518-8787.2023057004918)
Supplement: Translation

# Translation: Sintomas depressivos na infância interferem na inteligência na idade adulta?

**Authors:** Janielle Ferreira de Brito Lima, Raina Jansen Cutrim Propp Lima, Mônica Araújo Batalha, Antônio Augusto Moura da Silva, Marizélia Rodrigues Costa Ribeiro, Rosângela Fernandes Lucena Batista

**Affiliations:** I Brazil Universidade Federal do Maranhão. Departamento de Saúde Pública. São Luís, MA, Brazil.

**Keywords:** Criança, Adulto, Depressão, Inteligência

## Abstract

**OBJETIVO:**

Investigar os efeitos dos sintomas depressivos na infância no desenvolvimento intelectual do adulto jovem.

**MÉTODOS:**

Estudo realizado com uma coorte de nascimentos de São Luís, Maranhão, Brasil, composta por 339 participantes avaliados entre 7 e 9 anos e entre 18 e 19 anos. Utilizou-se modelagem de equações estruturais (escolaridade do adulto jovem, sexo, raça/cor) e variáveis da infância (estado nutricional, sintomas depressivos, função cognitiva, escolaridade do chefe da família e da mãe, renda familiar). Além disso, ocupação do chefe da família, idade da mãe e presença de companheiro foram testadas como determinantes do quociente de inteligência (QI) dos adultos.

**RESULTADOS:**

A presença de sintomas depressivos na infância gerou redução de 0,342 no desvio-padrão (DP) e -3,83 pontos no QI médio dos adultos (valor de p < 0,001). A função cognitiva na infância apresentou efeito total e direto positivo (coeficiente padronizado [CP] = 0,701; valor de p < 0,001) sobre o QI, elevando 7,84 pontos a cada aumento do nível. Identificou-se efeito indireto positivo do estado nutricional infantil (CP = 0,194; valor de p = 0,045), escolaridade do chefe da família (CP = 0,162; valor de p = 0,036) e da mãe da criança, este último mediado pela função cognitiva na infância (CP = 0,215; valor de p = 0,012) sobre o QI dos jovens.

**CONCLUSÃO:**

A presença de sintomas depressivos na infância gerou efeito negativo de longo prazo sobre a inteligência, reduzindo a pontuação do QI na idade adulta.

## INTRODUÇÃO

O desenvolvimento das habilidades cognitivas, representadas principalmente pela inteligência e desempenho acadêmico, tem sido amplamente estudado devido ao seu impacto em resultados socialmente relevantes como capacidade de liderança, sucesso no trabalho e vida social^1,2^.

Embora a inteligência seja fortemente influenciada pela hereditariedade, sabe-se que a estimulação parental, a educação e o estado nutricional e psicológico também afetam o seu desenvolvimento^3^. Entre essas variáveis, a saúde mental de crianças e adolescentes tem sido alvo de muitos estudos^4^ devido à elevada prevalência mundial de transtornos mentais nessa faixa etária nos últimos anos, em especial a depressão, que foi identificada em 6,2% dos indivíduos entre 5 a 17 anos em 38 países^4^. No Brasil os transtornos depressivos têm sido os mais prevalentes entre os problemas mentais que acometem crianças e adolescentes^5,8^.

Existem evidências de que sintomas de internalização e capacidade cognitiva são variáveis inter-relacionadas no desenvolvimento infantil até a adolescência^6^. Contudo, estudo realizado com crianças canadenses não encontrou evidências significativas de que a presença de sintomas de internalização interferiu no desempenho acadêmico na adolescência, que está diretamente relacionado ao desenvolvimento intelectual. O único efeito significativo foi uma associação positiva da internalização com o desempenho acadêmico dos 4 aos 11 anos^9^.

As alterações provocadas pelos sintomas depressivos normalmente acarretam prejuízo significativo na vida da criança, afetando o seu comportamento em casa, na escola e com os amigos. A queda do rendimento escolar é um dos primeiros indicadores de depressão em crianças, além do desenvolvimento do quadro de disforia, isolamento e tristeza^10^.

Ainda não estão claros o modo como os sintomas depressivos podem interferir no desenvolvimento intelectual até a idade adulta, os caminhos da associação entre essas variáveis e a forma como uma variável influencia a outra. Também é preciso entender se as dificuldades de uma causam dificuldades na outra ou se elas se relacionam apenas porque compartilham causas.

Assim, objetiva-se com este estudo responder às seguintes questões: a presença de sintomas depressivos na infância tem efeito direto na inteligência do adulto? Variáveis socioeconômicas dos pais durante a infância têm efeito maior sobre a inteligência do adulto que a presença de sintomas depressivos na infância? A associação entre os sintomas depressivos na infância e a inteligência na idade adulta é mediada pela cognição na infância?

## MÉTODOS

### Delineamento do Estudo

Trata-se de um estudo de coorte realizado com indivíduos nascidos na cidade de São Luís, Maranhão, Brasil, envolvendo dois períodos diferentes: infância e idade adulta. Esta coorte faz parte da pesquisa “Determinantes ao longo do ciclo vital da obesidade, precursores de doenças crônicas, capital humano e saúde mental”, desenvolvida pela Universidade Federal do Maranhão (UFMA), pela Faculdade de Medicina de Ribeirão Preto da Universidade de São Paulo (FMRP/USP) e pela Universidade Federal de Pelotas (UFPel).

### População e Amostra do Estudo

A primeira fase da coorte foi iniciada ao nascimento em dez hospitais públicos e privados da cidade, no período de março de 1997 a fevereiro de 1998, incluindo 96,3% dos nascimentos do período por meio de amostragem sistemática com estratificação proporcional de acordo com o número de nascimentos em cada maternidade de um em cada sete partos. Foram excluídos partos múltiplos, natimortos e gemelares. A amostra final totalizou 2.443 nascimentos^11^.

A segunda fase ocorreu quando as crianças estavam com 7 a 9 anos de idade, em 2005 e 2006, por meio de delineamento complexo de amostragem, utilizando a variável peso ao nascer para definir a amostra necessária para a avaliação na idade escolar. A amostra final totalizou 805 crianças nessa fase, sendo 673 acompanhadas desde o nascimento e 132 crianças nascidas entre 1997 e 1998 incluídas na coorte retrospectiva^8^.

A terceira fase ocorreu de janeiro a novembro de 2016, quando os sujeitos estavam com 18–19 anos de idade. Dos 805 participantes da etapa anterior, 339 participaram desse seguimento. Nessa fase, foram investigadas, entre outras, situação socioeconômica e demográfica, renda, hábitos de vida, habilidades cognitivas e doenças mentais^12^. Foram incluídos no estudo os sujeitos que, no momento da avaliação, se submeteram aos testes de saúde mental, função cognitiva na infância e desenvolvimento intelectual na idade adulta.

### Coleta de Dados

Na fase escolar, um questionário padronizado foi aplicado aos pais ou responsáveis pelas crianças contendo questões demográficas. Peso e altura foram medidos, com as crianças descalças e com roupas leves, utilizando balança de precisão calibrada periodicamente e um antropômetro^8^. Para avaliar a função cognitiva na infância, foi aplicado o teste de matrizes progressivas coloridas de Raven (MPCR), que avalia aspectos gerais da inteligência^13^; e teste de desenho da figura humana (DFH), que avalia a maturidade emocional e o desenvolvimento psicomotor^14^. Para avaliar a presença de sintomas depressivos na infância, utilizou-se o Inventário de Depressão Infantil^15^.

**Figura:** Diagrama de caminhos, com coeficientes padronizados, da associação de sintomas depressivos na infância com o quociente de inteligência de adultos jovens da Coorte do Consórcio RPS de São Luís. São Luís (Maranhão), Brasil, 2004–2016.

cog: cognição na infância; comp: presença de companheiro; cornc: raça/cor da criança; deprec: sintomas depressivos na infância; desn2: desnutrição; DFH: desenvolvimento cognitivo na infância mensurado pelo desenho da figura humana; esca2: escolaridade do adulto jovem; escc: escolaridade do chefe da família durante a infância; escolm: escolaridade materna durante a infância; idademae: idade da mãe da criança; ocupac: ocupação do chefe da família durante a infância; QI: quociente de inteligência do adulto jovem; raven: desenvolvimento cognitivo na infância mensurado pelo teste das matrizes progressivas coloridas de Raven; rendfam: renda familiar durante a infância; sexo: sexo.

A inteligência na idade adulta foi avaliada pela escala de inteligência de Wechsler para adultos (WAIS III), que retrata uma medida pontual do nível de inteligência. Para verificação dos resultados, inicialmente, foram analisados os dados brutos das escalas verbal e de execução. A partir da soma da pontuação de todos os subtestes, os resultados brutos foram convertidos em ponderados e analisados pela tabela correspondente ao manual WAIS III. A obtenção do quociente de inteligência (QI) total ocorreu pela soma dos valores brutos das escalas verbal e de execução, e pela análise dos valores na tabela por idade, refletindo os níveis de inteligência dos participantes^16^. Nos casos em que o participante apresentava alguma condição que impossibilitasse a realização do teste, este não foi aplicado.

### Variáveis

A variável dependente foi o QI do adulto jovem, obtida por meio da aplicação da WAIS-III. Para o modelo teórico testado, a variável foi tratada como numérica contínua. Para efeito de classificação pode ser categorizada como: inferior (≤ 89 pontos); médio (90 a 109 pontos); superior (≥ 110 pontos)^16^.

As variáveis explanatórias observadas na infância foram: sexo (feminino; masculino), raça/cor (preta/negra; parda/amarela/oriental; branca); estado nutricional: baixo peso (< 17 kg/m^2^) ou adequado (> 17 kg/m^2^)^8^; sintomas depressivos: ausentes (≥ 17 pontos) ou presentes (< 17 pontos)^15^; escolaridade do chefe da família e da mãe em anos completos (0–4 anos; 5–8; 9 ou mais); renda familiar em salários mínimos (numérica contínua); ocupação do chefe da família (manual não qualificado e desempregado; manual qualificado e semiqualificado; não manual); idade da mãe (numérica contínua); presença de companheiro (não; sim).

A variável função cognitiva na infância foi construída a partir das variáveis função cognitiva mensurada pelo teste MPCR, tratada como variável categórica: abaixo da média (0–25); média (26–74); acima da média (75–100)^13^ e função cognitiva mensurada pelo teste DFH, tratada como variável categórica: abaixo da média (< percentil 25); média (percentil 25); acima da média (> percentil 25)^14^. A variável escolaridade do adulto jovem foi tratada como categórica (0–4 anos; 5–8; 9 ou mais).

### Análise Estatística

A análise descritiva dos dados foi realizada utilizando a versão 14 do programa Stata (StataCorp., CollegeStation, Estados Unidos da América). As variáveis categóricas foram apresentadas através de frequências absolutas e relativas, as variáveis numéricas por média e desvio-padrão.

Utilizou-se modelagem de equações estruturais^17^ para investigar a associação dos sintomas depressivos na infância com as covariáveis e seus efeitos sobre a inteligência na idade adulta. Construiu-se um modelo híbrido, composto por análise fatorial confirmatória utilizada para construção da variável latente função cognitiva na infância e por análise de caminhos utilizada para analisar os efeitos dos sintomas depressivos na infância no QI na idade adulta e estimar as relações lineares entre as variáveis. Para isso utilizou-se a versão 7 do software Mplus.

De acordo com o modelo teórico proposto, as variáveis socioeconômicas da família, observadas durante a infância do participante, ocuparam a posição mais distal e indicavam a presença de sintomas depressivos, o estado nutricional e a função cognitiva da criança, que determinaram a escolaridade do adulto jovem e seu QI (Figura).

O estimador utilizado foi o dos mínimos quadrados ponderados robustos ajustados pela média e variância (*weighted least squares mean and variance adjusted* – WLSMV), empregado para análises de variáveis categóricas observadas. A parametrização teta (θ) foi utilizada para controlar as diferenças de variâncias residuais.

Os dados faltantes foram imputados nas variáveis pelo software Mplus, com base nas variáveis anteriores a ela no modelo teórico, utilizando análise de frequências e análise bayesiana^18^. Com a realização da imputação, os dados faltantes não prejudicaram o resultado.

Para avaliar o ajuste do modelo, considerou-se bom o seguinte: a) valor de p > 0,05 para o teste do qui-quadrado (χ^2^); b) valor do *root mean square error of approximation* (RMSEA) < 0,05 e limite superior do intervalo de confiança de 90% < 0,08; c) valores superiores a 0,95 para o comparative fit index (CFI) e o Tucker-Lewis index (TLI); e d) valores *weighted root mean square residual* (WRMR) < 1^17^.

Na análise das estimativas padronizadas para construção da variável latente cognição na infância, considerou-se carga fatorial > 0,5 com valor de p < 0,05 como indicativos de que a correlação entre a variável observada e o construto é moderadamente alta em magnitude^17^.

Utilizou-se o comando *modindices* para obtenção de sugestões de alterações das hipóteses iniciais^18^, entretanto, não houve propostas plausíveis do ponto de vista teórico. No modelo final foram avaliados os efeitos totais, diretos e indiretos da variável latente e das observadas. Julgou-se haver efeito quando valor de p < 0,05.

Para interpretar os efeitos das variáveis no QI dos adultos jovens, multiplicou-se o valor do coeficiente padronizado do efeito total obtido no modelo estrutural pelo desvio-padrão do QI.

### Aspectos Éticos

O estudo atendeu aos critérios da Resolução 466/2012^19^, do Conselho Nacional de Saúde do Brasil. Os responsáveis pelos sujeitos assinaram o termo de consentimento livre e esclarecido (TCLE). Foi facultada a desistência sem qualquer prejuízo para o entrevistado, em qualquer etapa da pesquisa. Os projetos e os TCLE das duas fases da pesquisa foram aprovados pelo Comitê de Ética em Pesquisa do Hospital Universitário da Universidade Federal do Maranhão: Parecer Consubstanciado nº 60, de 18 de abril de 2005, e Parecer Consubstanciado nº 1.302.489, de 29 de outubro de 2015.

## RESULTADOS

Na amostra de 339 adultos jovens, 55,16% eram do sexo feminino, 67,85% pardos/amarelos/orientais e 83,78% apresentavam 5 a 8 anos de estudo (Tabela 1). A média do QI dos participantes foi 99,45 ± 11,19 pontos. A maioria (60,47%) atingiu resultados classificados como médios, sendo o desempenho mais baixo 70 e o mais alto 137 pontos.

**Tabela 1:** Características socioeconômicas, demográficas, familiares, presença de sintomas depressivos e desenvolvimento cognitivo e quociente de inteligência dos adultos jovens da Coorte do Consórcio RPS de São Luís. São Luís (Maranhão), Brasil, 2004–2016.

Variáveis	n (%)
Da criança	
Sexo	
Feminino	187 (55,16)
Masculino	152 (44,84)
Raça/cor	
Preta	30 (8,85)
Parda/amarela/oriental	230 (67,85)
Branca	79 (23,30)
Desnutrição^a^	
Sim	25 (7,40)
Não	312 (92,31)
Desenvolvimento cognitivo (MPCR)^a^	
Abaixo da média	88 (25,96)
Médio	150 (44,25)
Acima da média	100 (29,50)
Desenvolvimento cognitivo (DFH)^a^	
Abaixo da média	102 (30,09)
Média	192 (56,64)
Acima da média	42 (12,68)
Sintomas depressivos^a^	
Ausente	277 (81,71)
Presente	62 (18,29)
Da família durante a infância	
Escolaridade da mãe (anos)^a^	
0–4	45 (13,27)
5–8	89 (26,25)
≥ 9	198 (58,41)
Presença de companheiro	
Não	70 (20,71)
Sim	269 (79,29)
Escolaridade do chefe da família (anos)^a^	
0–4	106 (31,27)
5–8	71 (20,94)
≥ 9	143 (42,18)
Renda familiar (em salários mínimos)^a^	
< 1	66 (19,47)
1 < 4	230 (67,85)
≥ 4	27 (7,96)
Ocupação do chefe da família^a^	
Manual não qualificado e desempregado	196 (56,64)
Manual qualificado e semiqualificado	100 (29,50)
Não manual	45 (13,27)
Do adulto jovem	
Escolaridade (anos)	
0–4	18 (5,31)
5–8	284 (83,78)
≥ 9	37 (10,91)
Quociente de inteligência (QI)	
Abaixo da média	63 (18,58)
Médio	205 (60,47)
Acima da média	71 (20,94)
Total	339 (100)

MPCR: teste de matrizes progressivas coloridas de Raven; DFH: teste do desenho da figura humana.

^a^ Excluídos os ignorados.

Durante a infância, 92,31% dos participantes do estudo apresentaram estado nutricional adequado e 18,29% manifestaram sintomas depressivos. De acordo com o teste das MPCR, 44,25% apresentaram função cognitiva média nessa fase e, de acordo com o DFH, 56,64%. A média de idade das mães dos participantes nessa fase foi de 23,15 ± 5,22 anos. A maioria delas convivia com companheiro (79,29%) e apresentava 9 ou mais anos de estudo (58,41%). No que se refere aos chefes da família, 56,64% estavam desempregados ou desempenhavam ocupação manual não qualificada e 42,18% apresentaram 9 ou mais anos de estudo. A renda de 67,85% das famílias estava entre um e menos que quatro salários mínimos (Tabela 1).

O modelo proposto para investigar os caminhos da associação entre a presença de sintomas depressivos na infância e a inteligência na idade adulta apresentou bom ajuste para os indicadores RMSEA, CFI, TLI e WRMR (Tabela 2) e não houve sugestões plausíveis de modificação.

**Tabela 2:** Índices de ajuste do modelo para desenvolvimento intelectual de adultos jovens da Coorte do Consórcio RPS de São Luís. São Luís (Maranhão), Brasil, 2004–2016.

Índices de ajuste	Modelo^a^
χ^2b^	265.810
Graus de liberdade	63
Valor de p	< 0,001
RMSEA	0,018
IC90%	0,000–0,049
Valor de p	0,957
CFI	0,985
TLI	0,968
WRMR	0,608

RMSEA: *root mean square error of approximation*; CFI: *comparative fit index*; TLI: *Tucker-Lewis index*; WRMR: *weighted root mean square residual*; IC: intervalo de confiança.

^a^ Modelo final com melhor ajuste.

^b^ Teste qui-quadrado.

A análise fatorial para construção da função cognitiva na infância mostrou que as variáveis indicadoras apresentaram correlação com o construto, apresentando carga fatorial > 0,5 e valores de p < 0,001 (Tabela 3).

**Tabela 3:** Coeficiente padronizado, erro-padrão e valores de p do construto cognição na infância e dos efeitos diretos das variáveis indicadoras no quociente de inteligência dos adultos jovens na Coorte do Consórcio RPS de São Luís. São Luís (Maranhão), Brasil, 2004–2016.

Variáveis	Coeficiente padronizado	Erro-padrão	Valor de p
Variável latente			
Cognição na infância			
Desenvolvimento cognitivo (DFH)	0,588	0,073	< 0,001
Desenvolvimento cognitivo (MPCR)	0,542	0,065	< 0,001
Efeitos diretos			
Quociente de inteligência			
Sintomas depressivos na infância	-0,171	0,122	0,16
Cognição na infância	0,662	0,141	< 0,001
Desnutrição na infância	0,046	0,097	0,633
Presença de companheiro	-0,072	0,067	0,28
Escolaridade materna	-0,025	0,079	0,75
Escolaridade do chefe da família	0,065	0,065	0,319
Escolaridade do adulto jovem	0,088	0,152	0,562
Cognição na infância			
Sintomas depressivos na infância	0,205	0,128	0,11
Desnutrição na infância	0,276	0,125	0,027
Idade da mãe	0,088	0,079	0,263
Escolaridade da mãe	0,325	0,095	0,001
Presença de companheiro	0,059	0,092	0,523
Renda familiar na infância	-0,06	0,093	0,524
Escolaridade do chefe da família	0,067	0,122	0,58
Ocupação do chefe da família	-0,048	0,089	0,592
Sintomas depressivos na infância			
Sexo	0,014	0,083	0,861
Raça/cor	0,12	0,081	0,141
Renda familiar na infância	0,092	0,081	0,256
Idade da mãe	0,123	0,091	0,202
Escolaridade materna	-0,065	0,093	0,484
Escolaridade do chefe da família	0,116	0,091	0,202
Desnutrição na infância			
Renda familiar na infância	-0,066	0,187	0,726
Escolaridade materna	-0,103	0,143	0,475
Escolaridade do chefe da família	0,291	0,152	0,056
Ocupação do chefe da família	-0,021	0,118	0,861
Escolaridade do adulto jovem			
Sintomas depressivos na infância	-0,315	0,133	0,018
Cognição na infância	0,437	0,121	< 0,001
Renda familiar na infância	0,088	0,104	0,398
Ocupação do chefe da família	0,109	0,093	0,243
Escolaridade do chefe da família	0,056	0,093	0,55
Escolaridade materna	0,087	0,124	0,48
Presença de companheiro	0,054	0,073	0,459

MPCR: teste de matrizes progressivas coloridas de Raven; DFH: teste do desenho da figura humana.

A presença de sintomas depressivos na infância apresentou efeito total negativo de -0,342 DP sobre a média do QI na idade adulta (Tabela 4), que corresponde a -3,83 pontos (valor de p < 0,001). O desenvolvimento cognitivo na infância apresentou efeito total e direto positivo sobre o QI (Tabela 4), revelando um incremento de 0,701 DP na sua média (valor de p < 0,001), correspondente ao aumento de 7,84 pontos a cada elevação do nível de desenvolvimento cognitivo infantil.

**Tabela 4:** Coeficiente padronizado, erro-padrão e valores de p dos efeitos total, direto e indiretos dos sintomas depressivos, cognição e desnutrição na infância e escolaridade da mãe e do chefe da família sobre o quociente de inteligência de adultos jovens da Coorte do Consórcio RPS de São Luís. São Luís (Maranhão), Brasil, 2004–2016.

Caminhos	Coeficiente padronizado	Erro-padrão	Valor de p
Efeitos totais, diretos e indiretos			
Sintomas depressivos na infância			
Efeito total	-0,342	0,08	< 0,001
Efeito direto	-0,171	0,122	0,16
Efeito indireto	-0,171	0,103	0,096
Cognição na infância			
Efeito total	0,701	0,102	< 0,001
Efeito direto	0,662	0,141	< 0,001
Efeito indireto	0,039	0,062	0,531
Desnutrição na infância			
Efeito total	0,24	0,078	0,002
Efeito direto	0,046	0,097	0,633
Efeito indireto	0,194	0,097	0,045
Escolaridade do chefe da família			
Efeito total	0,227	0,057	< 0,001
Efeito total	0,065	0,065	0,319
Efeito direto	0,162	0,077	0,036
Escolaridade materna			
Efeito total	0,163	0,056	0,004
Efeito direto	-0,025	0,079	0,75
Efeito indireto	0,188	0,084	0,026
Via cognição na infância	0,215	0,086	0,012

O estado nutricional do participante na infância (CP = 0,240; valor de p = 0,002) e as escolaridades do chefe da família (CP = 0,227; valor de p < 0,001) e da mãe do participante nessa fase (CP = 0,163; valor de p = 0,004) apresentaram efeito total positivo, predominantemente indireto, sobre o QI na idade adulta (Tabela 4). O efeito indireto positivo da escolaridade materna sobre o QI foi mediado pela cognição na infância (CP = 0,215; valor de p = 0,012) (Tabela 4).

## DISCUSSÃO

Neste estudo, a presença de sintomas depressivos em crianças teve resultados negativos de longo prazo no desenvolvimento da inteligência, reduzindo a pontuação do QI na idade adulta. Esse efeito não foi mediado pela função cognitiva na infância, cuja repercussão sobre o QI foi direta. Foram observados efeitos indiretos positivos do estado nutricional do participante na infância, da escolaridade materna e do chefe da família na pontuação do QI na idade adulta. Adicionalmente, observou-se que a cognição na infância parece mediar o efeito positivo da escolaridade materna na pontuação do QI na idade adulta.

É possível que a redução do QI observada nos adultos jovens que apresentaram sintomas depressivos na infância seja explicada pelo prejuízo na capacidade de pensar, de concentração ou de tomada de decisões que acompanha esses sintomas nas crianças. Nessa faixa etária, a queda no rendimento escolar é um dos primeiros sinais de depressão e pode ser uma manifestação de déficit de atenção^10^. Neste estudo, os sintomas depressivos na infância apresentaram efeito direto negativo sobre a escolaridade dos adultos jovens. Uma outra possível explicação é que os sintomas depressivos detectados na infância podem persistir e ser intensificados até a vida adulta, gerando impactos ao longo da vida^20^. Estudos anteriores demonstraram que a alterações de saúde mental na infância e adolescência podem comprometer o desempenho acadêmico e o nível de educação atingido, e este último, por sua vez, pode impactar nas oportunidades de trabalho e na carreira desses indivíduos^21,22^.

O efeito direto da cognição na infância sobre a inteligência na idade adulta observado no presente estudo está em consonância com estudos anteriores, que revelam uma alta estabilidade cognitiva ao longo da vida^23,24^. Yu et al.^23^ observaram, usando um construto latente de inteligência, que houve estabilidade considerável na inteligência ao longo dos quatro períodos investigados: infância, pré-escolar, escolar e adolescência. Além disso, a progressão longitudinal da inteligência desde a infância até a adolescência foi completamente mediada pela inteligência do período anterior. Adicionalmente, dados de 588 indivíduos da Lothian Birth Cohort 1936 revelaram que a capacidade cognitiva aos 11 anos foi preditora da espessura cortical aproximadamente 60 anos depois e mostrou-se responsável por mais de dois terços da associação transversal entre inteligência e espessura cortical na velhice^24^.

Diversos estudos já evidenciaram a importância da situação socioeconômica da família no desenvolvimento e cognição na infância e ao longo da vida^25,26^. Nesse contexto, a escolaridade tem sido usada como um dos principais indicadores de status socioeconômico^25^. Um estudo realizado com dados de sete países (n = 15.297), incluindo o Brasil, revelou que a educação dos pais seria mais importante do que a riqueza familiar para moldar a inteligência de seus filhos (4–22 anos)^26^.

A relação entre a educação dos pais e a inteligência dos filhos pode ser explicada por fatores tanto genéticos quanto ambientais^25,27^. Pais com maior nível de escolaridade parecem investir mais dinheiro e tempo em seus filhos, resultando em melhores condições de saúde e educação^28^, além de oferecer um ambiente intelectualmente mais estimulante, que poderia resultar em desempenho superior em alguns testes^29^. Evidências provenientes de países de baixa e média renda apontam que a educação materna seria um importante fator protetor para o bem-estar e desenvolvimento infantil^30^, com efeito aparentemente superior ao da educação paterna^31^. Mães com maior nível de escolaridade parecem proporcionar para seus filhos maiores oportunidades de aprendizado, interação de maior qualidade e menor experiência de estresse parental^32^. Somado a esses fatos, o estímulo materno está entre os principais mecanismos que explicam a relação entre a educação materna e o desenvolvimento infantil^31,33^.

Em concordância com nossos achados, estudos recentes evidenciam que o desenvolvimento infantil também pode ser afetado pelas condições nutricionais da criança^34,35^. A nutrição adequada, em um período crítico como a infância, é fundamental para o funcionamento cerebral ao longo da vida^35^. Além disso, crianças com adequado estado nutricional conseguem interagir melhor com seus cuidadores e com o ambiente, fatores fundamentais para o seu pleno desenvolvimento^36^.

Uma limitação deste estudo foi a perda de seguimento dos sujeitos, especialmente durante a terceira fase, devido à dificuldade de localização dos adultos jovens, apesar de todas as estratégias de busca utilizadas. Com uma amostra maior, seria possível detectar outros efeitos de determinantes importantes.

Um ponto forte é o tipo do estudo. Estudos de coorte tem vantagens em relação à causalidade reversa e à possibilidade de acompanhamento da mesma população. Outro ponto é o método estatístico utilizado: modelagem de equações estruturais. Esta modelagem tem a vantagem de lidar simultaneamente com várias relações de dependência e pode representar variáveis latentes nessas relações, além de modelar o erro de medição no processo de estimativa^17^.

Este estudo demonstrou que o QI na idade adulta é diretamente influenciado pelos níveis de desenvolvimento cognitivo na infância. Durante a infância, a situação socioeconômica favorável pode influenciar positivamente o QI na idade adulta, porém a presença de sintomas depressivos pode influenciar negativamente.
